# Adsorption Capacity, Isotherm, Kinetics, and Thermodynamics Examinations on the Removal of a Textile Azo Dye by Local Natural Adsorbent

**DOI:** 10.1002/gch2.202500024

**Published:** 2025-04-10

**Authors:** Fatih Sevim, Ömer Laçin, Fatih Demir, Ömer Faruk Erkiliç

**Affiliations:** ^1^ Engineering Faculty Department of Chemical Engineering Ataturk University Erzurum 25100 Turkey

**Keywords:** adsorption, adsorption isotherm, adsorption kinetics, textile dye

## Abstract

The discharge of industrial wastewater containing toxic dyes has significantly increased, posing risks to human health and aquatic ecosystems. The growing demand for dyes in the textile industry has driven research into effective and economical removal methods. Adsorption is widely preferred due to its low cost, non‐toxic by‐products, and eco‐friendly nature. This study investigates the removal of Reactive‐Blue‐160 textile azo dye using a local natural clay mineral. The effects of contact time, pH, adsorbent dosage, and temperature on adsorption are examined, along with adsorbent characterization. Optimal conditions (pH 5.70, adsorbent dosage 2.0 g L⁻¹, contact time 60 min, and dye concentration 150 mg L⁻¹) achieve 93.05% removal. Characterization reveals a heterogeneous clay surface dominated by smectite and chlorite. The adsorption data are evaluated using isotherm and kinetic models, with Freundlich and pseudo‐second‐order providing the best fit. Thermodynamic analysis indicates spontaneous and endothermic adsorption, with a negative Gibbs free energy and a positive enthalpy change of 15.71 kJ mol⁻¹, confirming physical adsorption. These findings highlight the potential of natural clay minerals for dye removal, offering a sustainable solution for industrial wastewater treatment.

## Graphical abstract

Azo dyes, which adversely affect human health and the ecosystem and are very difficult to treat, should be removed before being discharged into the environment. For this purpose, a low‐cost, non‐toxic natural clay mineral is used for the removal of Reactive‐Blue‐160 azo dye. It is seen through literature comparisons that azo dye removal with local natural clay is successful.

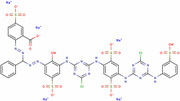

## 1 Introduction

Dyes are generally used to color products in the textile, pharmaceutical, cosmetic, plastic, leather, and rubber industries and cause toxicity due to the discharge of wastewater containing large amounts of dye.^[^ ^1^ ^]^ The toxicity levels vary depending on the variety of organic and inorganic compounds used in dyeing and other processes. When such wastewater is discharged into the environment, light transmittance in the aqueous environment decreases, photosynthetic activity is adversely affected, and toxicity increases. In addition, it causes many adverse effects in humans, such as cancer, reproductive and neurological disorders, severe allergies, rashes, and shortness of breath.^[^ ^2^, ^3^, ^4^ ^]^ One of the dyes widely used in the textile industry is azo dyes due to their excellent processing qualities, high photolytic stability, and resistance to microbial degradation. If these dyes are released into lakes and rivers without being removed, they will reduce the amount of dissolved oxygen in the water and will be highly toxic due to their carcinogenic and mutagenic properties.^[^ ^5^, ^6^, ^7^, ^8^, ^9^ ^]^ Azo dyes constitute the largest group of all synthetic colorants and have high discharge levels in wastewater from leather, pharmaceutical, cosmetic, and textile (silk, nylon, wool yarn, etc.) industries.^[^ ^10^ ^]^ The molecular structure of azo dyes is polyaromatic and contains multiple functional groups such as hydroxyl, amino, chlorotriazines sulfonate, and carboxylate. Due to the presence of these groups, the azo dye is very soluble in aqueous media and becomes mobile in soil. Therefore, it is very difficult to treat wastewater containing azo dyes.^[^ ^11^, ^12^, ^13^, ^14^ ^]^ Rapid and effective removal of azo dyes before discharge into water is a very important problem to not harm human health and the ecosystem. For this purpose, studies have been carried out to remove such dyes from textile wastewater using different methods such as coagulation/flocculation, ultrafiltration, membrane separation, electrolysis, chemical oxidation, and photocatalysis. However, these methods have some disadvantages; these include high treatment costs, rapid fouling of the membrane, sludge production, formation of toxic by‐products during the process, etc.^[^ ^15^ ^]^

Today, the adsorption process is used more widely than other methods in the removal of dyes due to its ease of use, low cost, not forming sludge and toxic intermediates, and environmentally friendly structure.^[^ ^16^, ^17^, ^18^ ^]^ The most important advantage of adsorption is that it completely separates the dye from the wastewater without breaking it down. Therefore, removing dyes also reduces the risk of cancer.^[^ ^19^, ^20^ ^]^ Activated carbon is widely used as an adsorbent in the adsorption process because it has a high surface area, microporosity, adsorption capacity, and surface reactivity. However, its high cost and difficulties encountered in its regeneration have directed researchers to more economical alternative adsorbents. To solve this problem, low‐cost adsorbents have also been investigated,^[^ ^21^, ^22^, ^23^, ^24^, ^25^ ^]^ but most of them have been subjected to chemical activation. Therefore, the application of natural and unprocessed adsorbents for the removal of azo dyes is missing in the literature.^[^ ^26^ ^]^

A typical example of natural and unprocessed adsorbents is clay minerals, which are used in the removal of dyes from wastewater. Since clay minerals are hydrated alumina silicates with colloidal particles smaller than 2 µm, they have high adsorption capacity, low cost, environmental friendliness, and a heterogeneous surface.^[^ ^27^ ^]^

Clay minerals also include other minerals such as calcite, feldspar, and quartz. On the surface of minerals, ions such as Ca^2+^, Mg^2+^, K^+^, H^+^, Na^+^, NH_4_^+^ and NO_3_^−^, SO_4_^2−^, PO_4_^3−^, Cl^−^ can easily exchange places with other ions in the environment.^[^ ^28^ ^]^

The novelty of this study is to propose a low‐cost and effective adsorbent for toxic azo dyes that have a wide range of applications and are difficult to remove from wastewater. Thus, the removal of azo dye will be greatly reduced in time and cost. In this study, the adsorption of Reactive‐Blue‐160 (RB160) dye found in textile industry wastewater by local natural and untreated green clay mineral (NGCM) was investigated. NGCM was characterized in terms of structure and morphology using X‐ray diffractometry (XRD), electron microscope (SEM), thermogravimetry/differential thermal analysis (TG‐DTA) and Brunauer Emmett‐Teller (BET) method. Fourier transform infrared spectroscopy analysis (FTIR) was performed to observe the structure of RB160 azo dye. The effects of NGCM dosage, mixing speed, initial RB160 concentration, contact time, and solution pH on adsorption were investigated. In addition, using the results obtained from experimental studies, the most suitable isotherm and kinetic models and thermodynamic parameters for the adsorption process were determined.

## 2 Results and Discussion

### 2.1 NGCM Characterization

**Figure 1:**
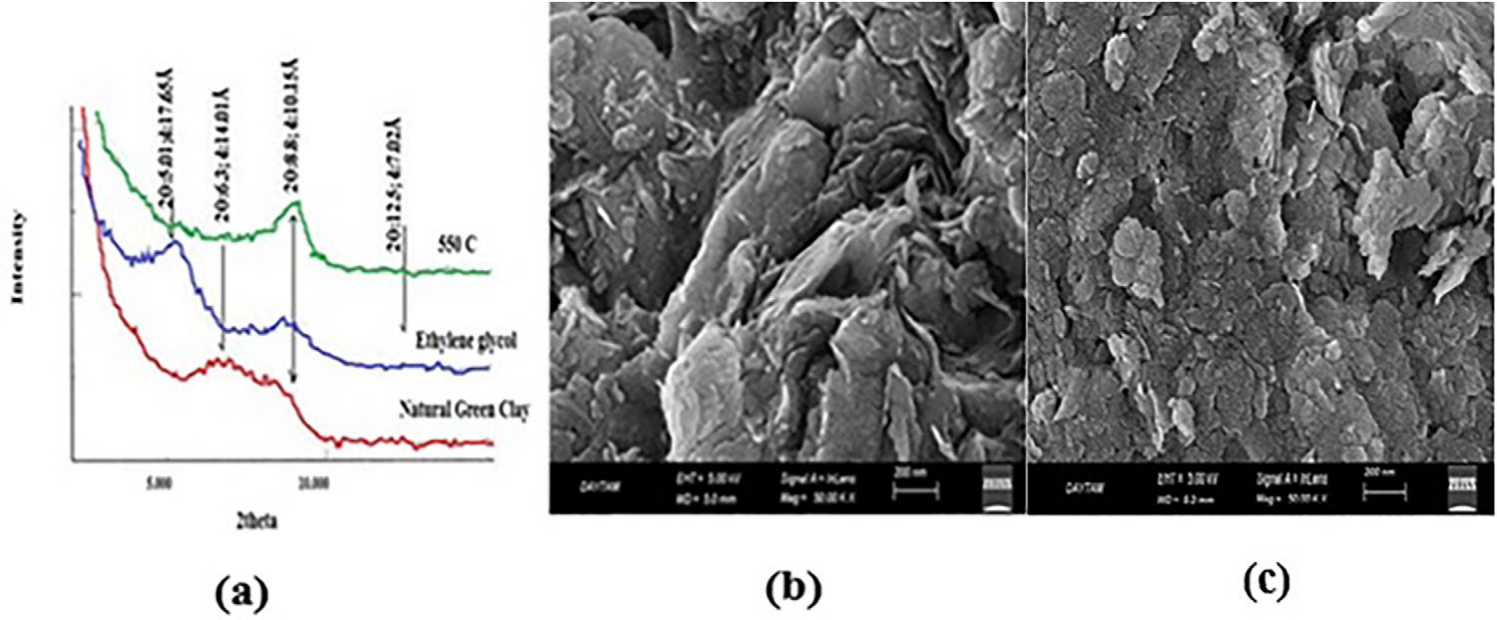
NGCM: a) XRD, b) SEM before adsorption, c) SEM after adsorption.

The X‐ray diffraction (XRD) pattern used to determine the crystal structure of the NGCM is shown in **Figure** **1**a. According to the XRD data of the ethylene glycol and natural clay samples, the peak corresponding to the mineral Smectite was measured to reach 17.65 Å at 14 Å. Moreover, the fracture angle of the ethylene glycol sample shifts to the left in the graph. According to the XRD analysis of clay fired at 550 °C, the mineral smectite, which lost its water, exhibited a peak at approximately 10 Å.

According to the XRD analyses of the illite minerals, the illite peak (10.15 Å) of the clay and natural clay samples saturated with ethylene glycol at 550 °C does not change.

Kaolinite: XRD analysis of the natural clay sample revealed a 2‐theta (12.5°) peak (*d* = 7.01 Å), while kaolinite has an amorphous structure when baked at 550 °C, and kaolinite peaks disappeared during XRD analysis.^[^ ^29^ ^]^

The surface of the adsorbent was imaged by scanning electron microscopy (SEM) before and after the adsorption process (Figure 1b,c). The images reveal that the natural green clay has a porous structure and that its outer surfaces have a heterogeneous structure with indentations and protrusions (Figure 1b). An examination of the SEM image of natural green clay after adsorption reveals that RB160 is adsorbed on natural green clay (Figure 1c).

**Figure 2:**
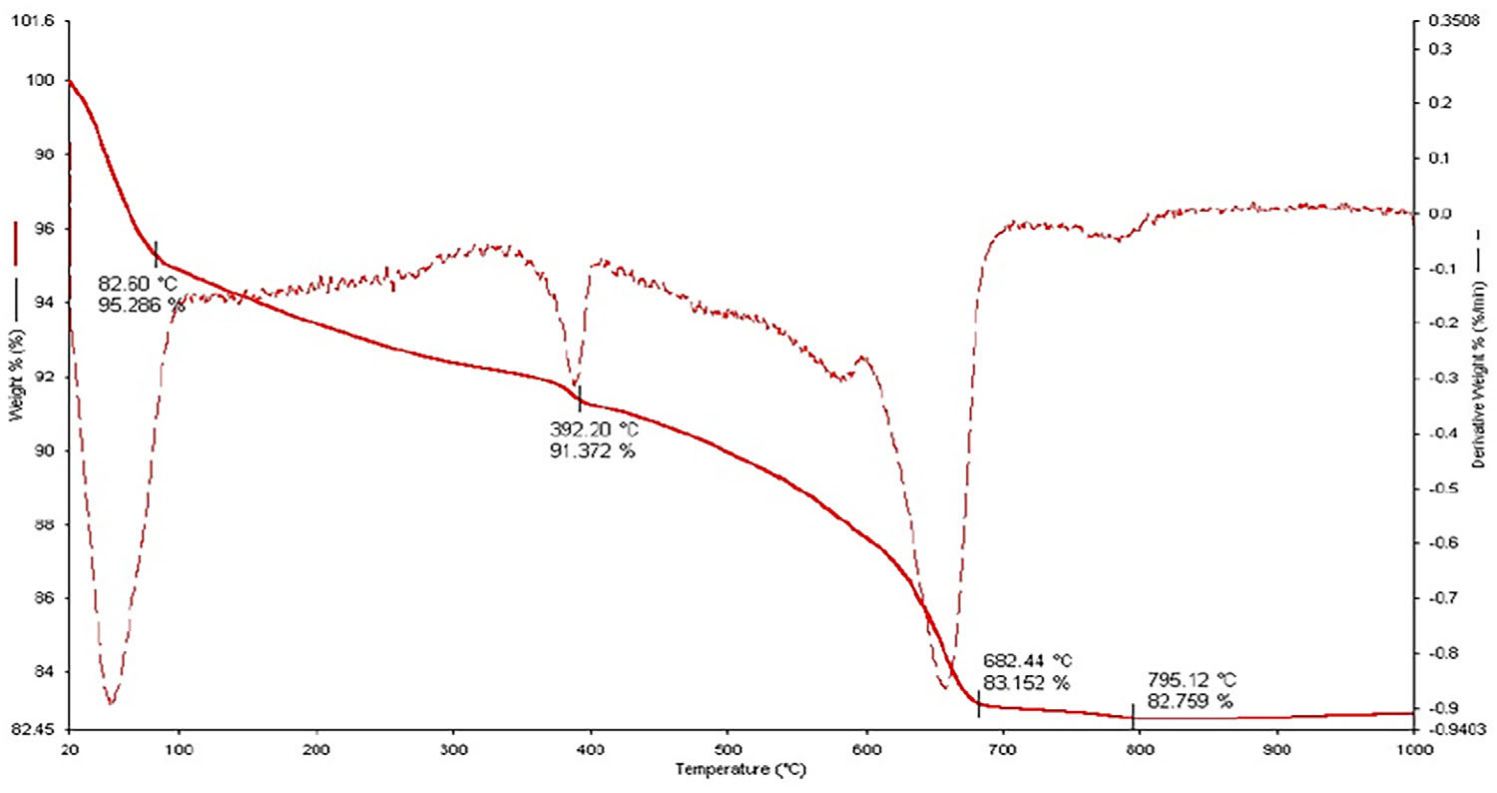
TG‐DTA analysis of the NGCM.

TG‐DTA‐ analysis of the NGCM sample revealed that the clay lost free water in the range of 80–100 °C (**Figure** **2**). The clay sample lost the crystalline water between the clay layers at 100–300 °C and above 300 °C. Dehydroxylation occurred in the clay sample due to endothermic reactions. Due to dehydroxylation, the red clay lost its structural water. Figure 2 shows the dehydroxylation peaks of illite minerals at 390 °C and montmorillonite minerals at 682 °C in natural green clay adsorbents.^[^ ^30^, ^31^, ^32^ ^]^

For NGCM pores, N_2_ adsorption/desorption analysis was performed at 75.9 K. As a result of the analysis, an isotherm with both type I and type IV properties was formed for NGCM. A steep increase was observed at relative pressures lower than 0.01, which is a typical feature of micropores. At higher relative pressures, desorption hysteresis and a mesoporous structure were observed. Brunauer‐Emmett‐Teller (BET) analysis determined that the surface area of ​​NGCM was 83.94 m^2^ g^−1^, pore volume was 0.10 cm^3^ g^−1,^ and average pore diameter was 4.57 nm.

**Figure 3:**
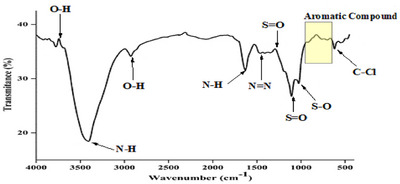
The FTIR spectra of the RB160.

The FTIR spectra of the RB160 is given in **Figure** **3**, and the results obtained from the spectra are presented in **Table** **1**. According to the results obtained, it can be said that RB160 is an aromatic substance containing functional groups such as hydroxyl, amino, chlorotriazine, sulfonate, and carboxylate. FTIR results confirmed the open structural formula of the RB160 azo dye in Figure 11.

**Table 1:** FTIR spectrum results of the RB160.

Wavenumber (cm^−1^)	Functional group
3739 3430 2932 1673 1463 1317 1127 1029	─OH stretch vibration
	N─H primary amin stretch vibration
	O─H carboxylate stretch vibration
	N─H amin stretch vibration
	N═N nitrosamine stretch vibration
	S═O stretch vibration (from SO_3_ groups)
	S═O stretch vibration (from SO_3_ groups)
	S─O sulfoxide stretch vibration
900‐700	Aromatic structure
637	C─Cl stretch vibration

### 2.2 Adsorption Equilibrium Studies

#### 2.2.1 Effect of Contact Time and Initial RB160 Concentration on the Adsorption Capacity

**Figure 4:**
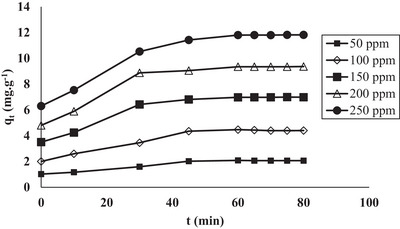
Determination of the equilibrium time (free pH 5.70, adsorbent dosage 2.0 g L^−1^, temperature 20 °C, stirring speed 225 rpm, 50–250 ppm RB160 concentration).

The effect of the initial RB160 concentration on the NGCM is shown in **Figure** **4**. This figure shows that the rate of adsorption is high at the beginning of the process and then becomes stationary at equilibrium. The high adsorption rate at the beginning can be explained by the elevated number of active zones on the adsorbent surface, after which the reaction speed decreases due to the reduction in these zones. The adsorption capacity increased with increasing initial NGCM concentration. This increase demonstrated that the dye ions between the liquid and the solid overcome the resistance to mass transfer and are due to the higher concentration gradient.^[^ ^33^, ^34^, ^35^ ^]^

When the data obtained for RB160 adsorption on the NGCM adsorbent at different initial concentrations were analyzed, the equilibrium time was 60 min. For this reason, the equilibrium time was taken as 60 min for all subsequent adsorption experiments.

#### 2.2.2 Effect of Initial Solution pH on the Adsorption Capacity

**Figure 5:**
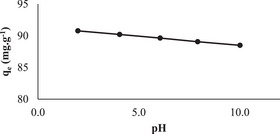
Determination of the suitable pH (contact time 60 min, adsorbent dosage 2.0 g L^−1^, temperature 20 °C, stirring speed 225 rpm, and RB160 concentration 150 mg L^−1^).

In this experiment, the adsorbent amount, contact time, temperature and concentration were held constant. The effect of pH on the adsorption of RB160 dye on NGCM was investigated, and the results are shown in **Figure** **5**.

The adsorption capacities at pH 2‐4‐6‐8‐10 were examined under constant conditions.

As shown in Figure 5, there was not much change in the adsorption capacities with increasing pH.

This can be explained by the fact that due to the sulfonate groups in the RB160 dye (pK ≈ 1.0),^[^ ^36^, ^37^, ^38^ ^]^ a pH approaching the pK of sulfonate groups through ionic interactions between the adsorbent and cationic dye molecules creates competition between H‐bonds, charge‒charge repulsion interactions, and RB160 adsorption against H^+^ ions.

On the other hand, with an increase in pH, the deterioration in hydrogen bond interactions due to excess OH^−^ ions may be the reason for the observed low decrease in adsorption capacity. Since the change in adsorption capacity with increasing pH was negligible, 5.70 was used as the free pH in all the experiments.

#### 2.2.3 Effect of the Adsorbent Dosage on the Adsorption Capacity

**Figure 6:**
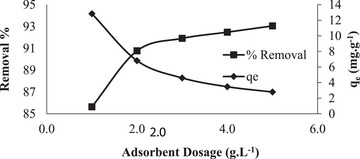
Effect of adsorbent dosage (contact time 60 min, temperature 20 °C, free pH 5.7, stirring speed 225 rpm, and RB160 concentration 150 mg L^−1^).

The NGCM adsorbent was added to 100 mL of 150 mg L^−1^ RB160 solutions at concentrations of 1, 2, 3, 4, and 5 g L^−1^. The pH, contact time, and temperature were kept constant. The removal percentage and adsorption capacity of RB160 are shown in **Figure** **6**. According to the data obtained from the experiments, when the dosage of adsorbent was 1–5 g L^−1^, the removal percentage increased from 85.64 to 93.05%. The increase in the removal percentage is due to the increase in the number of adsorption sites. Nevertheless, an increase in the dosage of adsorbent between 1 and 5 g L^−1^ is associated with a low removal rate. For this reason, the adsorption capacity decreased with increasing adsorbent dosage.^[^ ^39^ ^]^

#### 2.2.4 Temperature Effect

**Figure 7:**
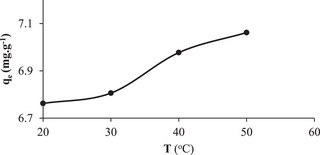
Effect of temperature change on RB160 adsorption (free pH 5.7, adsorbent dosage 2 g L^−1^, stirring speed 225 rpm, concentration of 150 mg L^−1^).

The adsorption capacity at different temperatures (20, 30, 40, 50 °C) for a concentration of 150 mg L^−1^ is shown in **Figure** **7**. The experiments were carried out with a fixed adsorbent dosage of 2 g L^−1^ and a stirring speed of 225 rpm at a free pH of 5.7. The results indicated that the uptake of the RB160 dye was favored at high temperatures. Adsorption may increase due to an increase in the rate of diffusion of the adsorbate molecules across the surface boundary layer and the presence of internal pores in the adsorbent particles.^[^ ^40^ ^]^ It was determined that temperature had a positive effect on the adsorbed amount, which demonstrated that the adsorption process was endothermic in nature.^[^ ^41^ ^]^

### 2.3 Analysis of the Adsorption Isotherms

The equilibrium correlations between the adsorbent and adsorbate are defined by adsorption isotherms.^[^ ^42^ ^]^ It is important to establish the most appropriate correlation for equilibrium curves to optimize the design of an adsorption system.^[^ ^43^ ^]^ The most commonly used isotherm models for aqueous solutions are the Langmuir, Freundlich, Temkin, and Dubinin‐Radushkevich isotherm models.

**Figure 8:**
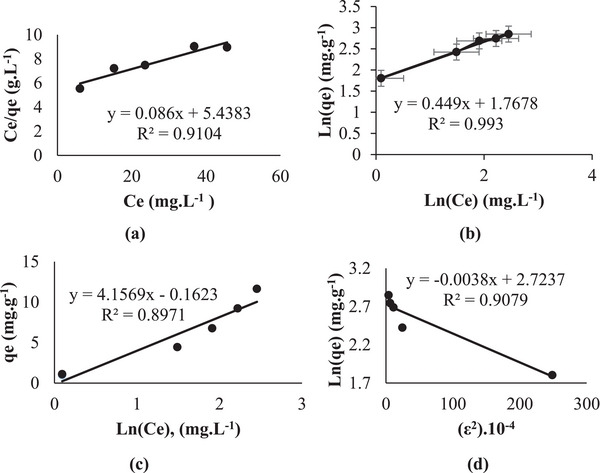
Isotherm curves for different isotherm models (a‐ Langmuir, b‐ Freundlich, c‐ Temkin, and d‐ Dubinin‐Radushkevich) (contact time: 60 min; free pH: 5.70; adsorbent dosage: 2.0 g L^−1^; temperature: 20 °C; stirring speed 225 rpm).

This experiment was carried out at a temperature of 20 °C. The four isotherm equations were applied to experimental equilibrium data for RB160 at 20 °C, an adsorbent dosage of 2 g L^−1^, and a free pH of 5.70. From Equation (1), the Langmuir isotherm parameters *K*_L_ and *q*_m_ were found from the intercept and slope of the linear relation between Ce/qe and Ce. The other isotherm constants in Equations ((3), (4), (5)) can be calculated in the same way. The calculated isotherm parameters for the adsorption of RB160 onto NGCM are shown in **Figure** **8**, and the details of the other parameters and correlation coefficients (*R* ^2^) are given in **Table** **2**.

**Table 2:** Parameters and correlation coefficients of the equilibrium isotherm models for the adsorption of RB160 onto NGCM at 20 °C.

Isotherm parameters	RB160 dye
Langmuir	
*q*_m_ (mg g^−1^)	11.63
*K*_L_(L mg^−1^)	2.13
*R*_L_	0.003
*R* ^2^	0.91
Freundlich	
*K*_f_ [(mg g^−1^)(L mg^−1^)^−1/^ *^n^*]	5.85
*N*	2.23
*R* ^2^	0.99
Temkin	
*K*_T_ (L mg^−1^)	1.04
*B*_T_	4.16
*R* ^2^	0.90
Dubinin‒Radushkevich	
*β*_DR_ (×10^−6^mol^2^ kj^−2^)	0.4
*q*_m_ (mg g^−1^)	15.23
*R* ^2^	0.91

When Table 2 and Figure 8 are examined, it can be said that the Freundlich isotherm model (*R* ^2^ = 0.99), which has the highest correlation coefficient, is the model that best fits the experimental data.

**Figure 9:**
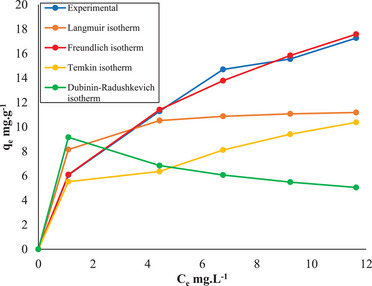
The comparison of isotherm curves.

Moreover, **Figure** **9** shows a comparison of the experimental isotherm curve with the curves obtained from these isotherm models. When this graph is examined, it is seen that the experimental and Freundlich isotherm curves overlap while the other curves deviate. This ultimately confirms that the experimental data fits the Freundlich isotherm model.

Additionally, the fact that the n value of the model parameters is greater than 1 indicates that the NGCM adsorbent can be used for the removal of RB160 dye. The Langmuir isotherm parameters reveal that the maximum monolayer adsorption capacity of the NGCM adsorbent at equilibrium is 11.63 mg g^−1^. Additionally, because the *R*_L_ parameter value (0.003) in this model is in the range of 0–1, this adsorption process is suitable.^[^ ^44^, ^45^ ^]^

### 2.4 Analysis of the Adsorption Kinetics

In adsorption processes, kinetics must be examined to obtain important information about the adsorption rate and adsorption mechanism. In kinetic studies of dye removal, the accepted mechanism in the literature includes the following steps: penetration of dye molecules from the solution to the adsorbent surface, diffusion of the dye from the pore surface into the pore, and interaction between dye molecules and reactive regions (chemical bonding, electrostatic interactions, ion exchange, hydrogen bonds, etc.).^[^ ^36^, ^46^ ^]^

**Figure 10:**
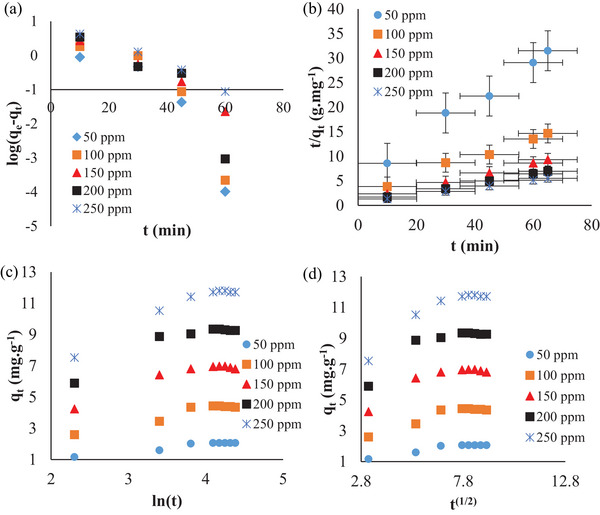
Kinetics curves for different kinetics models (a‐ Pseudo‐first‐order, b‐ Pseudo‐second‐order, c‐ Elovich, d‐ Intraparticle diffusion) (adsorbent dosage 2.0 g L^−1^, free pH 5.70, stirring speed 225 rpm and temperature 20 °C).

In light of this information, the suitability of the experimental data for the pseudo‐first‐order, pseudo‐second‐order, Elovich, and intraparticle diffusion kinetic models accepted in the literature was investigated. According to the pseudo‐first‐order kinetic model given in Equation (6), the graphs of log(q_e_‐q_t_) versus t are plotted in **Figure** **10**. The kinetic parameters k_1_ and q_e_ in this model were found from the intercept and slope of the linear relationship. The parameter values of the other kinetic models in Equations ((7), (8), (9)) were also calculated in the same way.

The suitability of the experimental data obtained from the adsorption process to the kinetic models is shown in Figure 10, and details regarding the parameter values and correlation coefficients (*R* ^2^) in the kinetic models are given in **Table** **3**. When Table 3 and Figure 10 are examined, it can be said that the pseudo‐second‐order kinetic model (*R* ^2^ = 0.99), which has the highest correlation coefficient, is the model that best fits the experimental data.

**Table 3:** Comparison of the kinetic models for different initial concentrations of RB160 at 20 °C.

Dye	*C*_0_	*q*_e,exp_	Pseudo‐first‐order			Pseudo‐second‐order OrderOrder		
			*k*_1_	*q*_e,cal_	*R* ^2^	*k*_2_	*q*_e,cal_	*R* ^2^
	50	2.06	0.17	2.21	0.80	0.03	2.06	0.99
RB160	100	4.43	0.17	5.12	0.81	0.01	4.42	0.99
	150	6.95	0.09	7.19	0.98	0.02	6.98	0.99
	200	9.34	0.15	10.06	0.81	0.01	9.35	0.99
	250	11.72	0.08	12.12	0.98	0.01	11.76	0.99

Dye	Co	Elovich			Intraparticle diffusion			
		*α*	*β*	*R* ^2^	*k*_dif_	*C*	*R* ^2^	
	50	0.58	2.15	0.94	0.16	0.73	0.90	
	100	1.56	1.08	0.93	0.32	1.75	0.88	
RB160	150	4.72	0.79	0.89	0.42	3.49	0.79	
	200	8.64	0.63	0.88	0.53	5.01	0.77	
	250	9.42	0.48	0.95	0.70	6.03	0.87	

**Figure 11:**
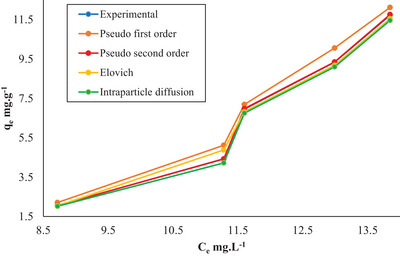
The comparison of experimental data with kinetic models.

In addition, **Figure** **11** shows a comparison of the experimental data with the curves obtained from the kinetic models. When this graph is examined, it is seen that the experimental data and the pseudo‐second‐order kinetic model curve overlap while the other curves deviate. This ultimately confirms that the experimental data fits the pseudo‐second‐order kinetic model.

### 2.5 Adsorption Thermodynamics

**Figure 12:**
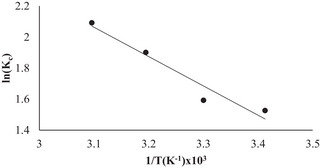
ln*K*_c_ versus 1/*T*.

The experiments were performed at temperatures ranging from 20–50 °C to determine the thermodynamic parameters of the adsorption processes. During the determination of the adsorption thermodynamics, parameters such as a free pH of 5.70, an initial concentration of 150 mg L^−1^ RB160 solution, a 225 rpm stirring speed, and an adsorbent dosage of 2.0 g L^−1^ were studied. The values of Δ*H*° and ΔS° can be found as the intercept and slope of the linear fit from the ln *K*_c_ versus 1/*T* plot (**Figure** **12**), respectively, and can be used to calculate Δ*G*° via Equation (13). All the calculated parameters are presented in **Table** **4**.

**Table 4:** Thermodynamic parameters for the adsorption of RB160 onto NGCM.

*T* [K]	Δ*H* ^○^ [kJ mol^−1^]	Δ*S* ^○^ [kJ mol^−1^]	Δ*G* ^○^ [kJ mol^−1^]
293	15.71	0.07	−4.80
303	15.71	0.07	−5.50
313	15.71	0.07	−6.20
323	15.71	0.07	−6.90

According to Table 4, the change in ∆*H*° (15.71 kJ mol^−1^ for NGCM) is positive and less than 20 kJ mol^−1^, indicating that the adsorption process occurs endothermically and physically with weak Van Der Waals bonds, respectively. Since dyestuffs are hydrated in water, they support the endothermic and physical achievement of the adsorption process. The positive change in ∆S° (0.07 J mol^−1^) indicates that the randomness at the solid–liquid interface increases during the adsorption of the RB160 dye to the active regions of the NGCM surface, and the dye has good affinity for the adsorbent.^[^ ^47^ ^]^

The fact that the ∆*G*° change is negative at all experimental temperatures shows that the adsorption reaction occurs spontaneously by the 2nd law of thermodynamics.^[^ ^48^ ^]^

### 2.6 Cost Analysis of NGCM

For the usability of NGCM as an economical adsorbent in large‐scale industrial applications, a production cost analysis was carried out considering chemical and energy costs. The cost analysis of RB160 per kg is given in **Table** **5**. The total production cost of NGCM was found to be 11.49 USD/to remove RB160.kg.^[^ ^2^ ^]^

**Table 5:** The cost analysis to remove RB160 per kg.

Name	Cost (USD)		Required amount	Cost (USD) of required amount
Sieved NGCM	1 kg	0.1143^a)^	100 kg	11.43
Muffle furnace (5000 W)	1 kWh	0.063	1 h	0.315
Magnetic stirrer (15W)	1 kWh	0.063	1 h	9.450×10^−4^
Spectrophotometer (80 W)	1 kWh	0.063	20 min	1680×10^−3^
Dirty water submersible pump (4 kW)	1 kWh	0.063	1 h	0.252
Total production cost				11.49

^a)^ This value was determined according to the cement price in Turkey for 2025.^[^ ^49^ ^]^

### 2.7 Comparison Study of NGCM on RB 160 Azo Dye Adsorptive Removal

Rapid and effective removal of azo dyes before discharge into water is a very important issue in order not to harm human health and ecosystem. In the removal of azo dyes with different adsorbents, the isotherm and kinetic models and maximum adsorption capacity of the previous studies and this study are compared in **Table** **6**. When Table 6 is examined according to maximum adsorption capacity values, it can be seen that they have low values ​​in almost all studies. This result confirms the difficulty of treatment of azo dyes mentioned in the introduction. When Table 6 is examined according to adsorbents, most of the adsorbents in other studies were activated and used. However, approximately similar adsorption capacity values ​​were achieved with the natural adsorbent used in this study. This result can be said that this study is especially advantageous in terms of economy. When Table 6 is examined according to isotherm and kinetic models, it can be seen that in other studies, Langmuir and Freundlich isotherm models and pseudo‐first and second order kinetic models are suitable. In the light of the data obtained from the experimental results in this study, the isotherm and kinetic models and the maximum adsorption capacity were found to be Freundlich, pseudo‐second order and 11.63 mg g^−1^, respectively. Similar results have been obtained when compared with the studies published in the literature.

**Table 6:** Comparison of this study with studies on the adsorption of azo dyes in the literature.

Adsorbents	Azo dyes	Isotherm models	Kinetics models	Capacity [mg g^−1^]	Refs.
Activated wheat husk	Yellow ME7 GL	Langmuir	−	0.46	[4]
Graphene oxide–chitosan nanocomposite	Acid blue 74	Langmuir	Pseudo‐second‐order	85.70	[5]
	Acid yellow 36	Langmuir	Pseudo‐second‐order	68.86	
Rice husk biomass activated carbon	Acid Yellow 36	Freundlich and Langmuir	Lagergren first order	86.9	[6]
Lignocellulosic waste biomass activated carbon	Amido Black 10B	Freundlich	Pseudo‐second‐order	4.033	[8]
Peanut husk biomass activated carbon	Yellow BG	Langmuir	Pseudo‐second‐order	25.9	[9]
Sugarcane bagasse Sugarcane bagasse (HCl‐treated)	Direct Violet 51	Langmuir	Pseudo‐second‐order	17.28 39.6	[10]
Anthracite	Acid yellow 42	Freundlich	Pseudo‐second‐order	47.00	[26]
Magnetic composite Fe_3_O_4_/CeO_2_	Acid black 210	Langmuir	Pseudo‐second‐order	93.00	[50]
Nanoparticles tin oxide	Congo red azo dye	Langmuir	Pseudo‐second‐order	48.30	[51]
NGCM	RB160	Freundlich	Pseudo‐second‐order	11.63	In this study

### 2.8 Adsorption Isotherm and Kinetic Models: Statistical Analysis

The statistical method was analyzed with nonlinear regression in Microsoft Excel program. Adsorption isotherm models are utilized to understand the equilibrium relationship between the adsorbent surface and the adsorbate. This study evaluated the compatibility of the Langmuir, Freundlich, Temkin, and Dubinin‐Radushkevich (D‐R) isotherm models using R^2^, P‐test, and F‐test values given in **Table** **7**.

**Table 7:** Isotherm models regression analysis.

Isotherm model	*R* ^2^	P‐test	F‐test
Langmuir	0.917	0.0068	45
Freundlich	0.992	0.0006	228
Temkin	0.889	0.0162	24
Dubinin–Radushkevich	0.906	0.0007	107

The Langmuir isotherm model assumes that adsorption occurs as a monolayer process on homogeneous surfaces and that there are no interactions between adsorption sites. In this study, the compatibility of the Langmuir model with the adsorption process was evaluated using *R* ^2^ = 0.917, *P* = 0.0068, and *F* = 45. The high *R* ^2^ value indicated that the model provides a certain degree of fit to the adsorption data. However, the relatively low F‐test value and the P‐test result being at the 0.01 level suggested that the model was statistically weaker compared to the Freundlich model. This indicates that the adsorption process was not strictly monolayer and that deviations from surface homogeneity may exist.

The Freundlich isotherm model assumes that adsorption occurs as a multilayer process on heterogeneous surfaces and that interactions may exist between adsorption sites. For this model, the obtained values were *R* ^2^ = 0.992, *P* = 0.0006, and *F* = 228. The high *R* ^2^ and F‐test values indicated that this model best explained the adsorption process. Additionally, the low P‐test value demonstrated the strong statistical significance of the model. The results suggest that the adsorption process took place on a heterogeneous surface and that the multilayer adsorption mechanism is predominant.

The Temkin isotherm model assumes that the heat of adsorption is evenly distributed across the surface and that there is a specific interaction between adsorbate molecules. The obtained values for this model, *R* ^2^ = 0.889, *P* = 0.0162, and *F* = 24, indicated that it described the adsorption process with lower accuracy compared to other isotherm models. In particular, the low F‐test value suggested that the model did not exhibit strong statistical significance. This finding implied that the heat of adsorption was not uniformly distributed and that the adsorption process did not occur on a homogeneous surface.

The Dubinin‐Radushkevich isotherm model is used to determine the physical or chemical nature of adsorption and plays a significant role in understanding the adsorption mechanism, particularly in porous materials. The obtained values for this model were *R* ^2^ = 0.906, *P* = 0.0007, and *F* = 107. The high *R* ^2^ and F‐test values indicated that the model effectively described the adsorption process. The strong fit of the Dubinin‐Radushkevich model suggested that the adsorption process may have a physical characteristic. This finding implied that the adsorption mechanism was not solely limited to chemical bonding but that physical interactions also played a crucial role.

Adsorption kinetics is a crucial parameter for understanding the rate of the adsorption process and its controlling mechanism. In this study, the pseudo‐first‐order, Pseudo‐second‐order, Elovich, and intraparticle diffusion models were evaluated, and model fitting was determined through statistical analyses given in **Table** **8**.

**Table 8:** Kinetic models regression analysis.

Kinetic model	*R* ^2^	P‐test	F‐test
Pseudo‐first‐order	0.993	0.0001010	768
Pseudo‐second‐order	0.999	<0.0000001	249802
Elovich	0.994	0.0000860	861
Intraparticle diffusion	0.998	0.0000007	20162

When Table 8 is examined; *R* ^2^ values ​​very close to 1, *p* values ​​less than 0.0001 and very large *F* values ​​indicate that all kinetic models are statistically significant and in agreement with experimental data. However, in the pseudo‐2nd order model, it can be said that it is the most suitable kinetic model because the p value is very small compared to other model values ​​and the F value is very large compared to other model values. This result also confirms the result in Section 2.4.

## 3 Conclusion

The adsorption of RB 160 azo dye, which is reported to be very difficult to remove from wastewater,^[^ ^11^, ^12^, ^13^, ^14^ ^]^ was studied with a local and natural clay (NGCM) adsorbent, which is thought to be economical and effective. In the adsorption process; adsorbent characterization, adsorption capacity, isotherm and kinetic models, thermodynamic parameters, cost analysis and comparison with other adsorbents used in azo dyes were investigated.

For NGCM characterization, SEM, XRD, TG‐DTA and BET analyses were performed and from the obtained results, it was determined that the adsorbent was alumina silicate containing smectite, kaolinite, illite and chlorite clay minerals, had a porous structure with heterogeneous surfaces, lost its structural water at 682 °C, had a surface area of ​​83.94 m^2^ g^−1^, a pore volume of 0.10 cm^3^ g^−1^ and an average pore diameter of 4.57 nm. From the FTIR spectra of the RB160 azo dye, it can be said that the adsorbate is an aromatic substance containing functional groups such as hydroxyl, amino, chlorotriazine, sulfonate, and carboxylate.

The effects of adsorbent amount, contact time, temperature, solution pH, and initial solution concentration on the adsorption process were investigated. In the effect of contact time, the adsorption equilibrium time was determined to be 60 min. In the effect of pH, although the removal of RB160 was 90.77% at the highest pH 2 and 88.49% at the lowest pH 10, since there was no effective change, the experiments were carried out at the free solution pH for economic purposes. Other conditions: temperature was 20 °C, initial solution concentration was 150 mg L^−1^ and the amount of adsorbent was determined as 2 g L^−1^. RB160 adsorption increased with increasing solution temperature, contact time, and initial dye concentration.

By comparing the correlation coefficient and the curves obtained from the experimental data and the models, it was decided that the most suitable isotherm and kinetic models for the adsorption process were the Freundlich isotherm and pseudo‐second order, respectively. According to the values ​​of the isotherm constants, it can be said that the surface of the adsorbent is heterogeneous and is suitable for use as an adsorbent.

The thermodynamic parameter values ​​of Δ*G*°, Δ*H* ^o,^ and Δ*S*° were calculated as −4.80 to −6.90, 15.71, and 0.07 kJ mol^−1^, respectively. According to these results, it can be said that the adsorption process was spontaneous as the Δ*G*° value was negative and endothermic reaction and physical adsorption as Δ*H*° value was positive and less than 20 kJ mol^−1^. By cost analysis, it was found that the removal cost of NGCM per kg of RB160 was approximately 11.49 US dollars.

In this study, the adsorption capacity, isotherm, and kinetic model were compared with the results of azo dye removal with different adsorbents in the literature. Due to the difficulty of azo dye purification, it was observed that the maximum adsorption capacity values ​​were low in almost all studies, including this study. However, while most of the adsorbents in other studies were activated and used, a similar capacity value was reached with the natural adsorbent in this study. Therefore, it can be said that this adsorption process is advantageous especially in terms of economy.

In recent years, due to the high CO_2_ emissions in cement production, geopolymer concrete studies have been carried out quite a lot by activating clay minerals containing alumina silicate with alkaline solutions instead of cement. It can be suggested that the NGCM used in this study, which contains alumina silicate, will also eliminate possible environmental risks if used as a geopolymer concrete after adsorption.^[^ ^52^, ^53^, ^54^, ^55^ ^]^

## 4 Experimental Section

### Clay Material

A natural green clay mineral (NGCM) taken from the Erzurum‐Oltu/Turkey region was used as an adsorbent. Mineral analysis of the NGCM sample is shown in **Table** **9**. **Table** **10** shows the quantitative analysis of the NGCM. The NGCMs were passed through a ‐200 mesh sieve and washed with deionized water before the experiments. The sample was then dried in an oven at 105 °C overnight.

**Table 9:** Mineral analysis of the natural green clay sample.

	NGCM [%]
Smectite	34
Kaolinite	18
İllite	22
Chlorite	26

**Table 10:** Chemical components of the natural green clay sample.

	NGCM [%]
Na_2_O	2.36
MgO	7.29
Al_2_O_3_	13.70
SiO_2_	45.12
K_2_O	2.61
CaO	7.48
TiO_2_	0.52
Fe_2_O_3_	5.62
LOI	13
SiO_2_/Al_2_O_3_	3.29
SiO_2_/Fe_2_O_3_	8.02
SiO_2_/MgO	6.18
SiO_2_/CaO	6.03

### Adsorbate

**Figure 13:**
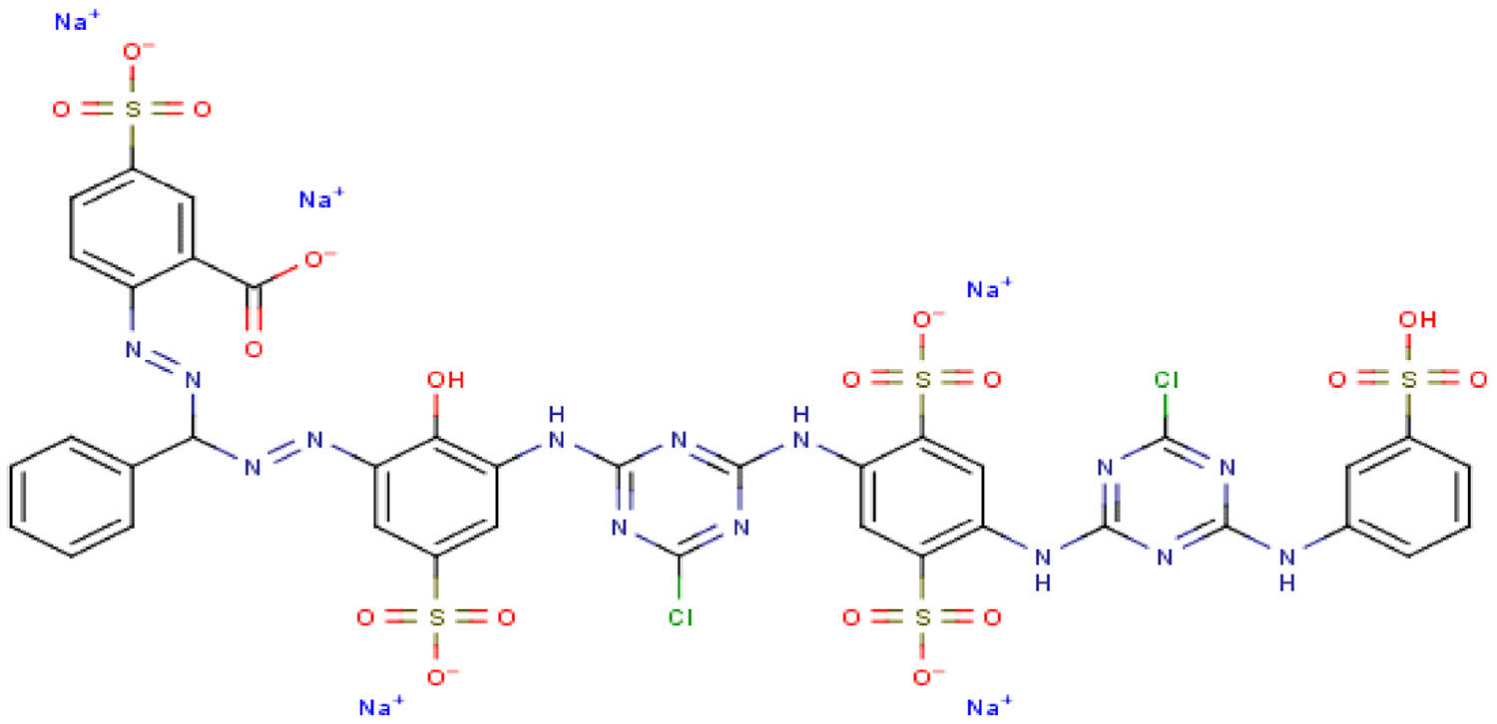
Structure of C.I. Reactive Blue 160.^[^ ^56^ ^]^

RB 160 azo dye was purchased from Alfa Chemical Company in Adana/Turkey with the brand Sigma‐Aldrich and 100% purity. RB 160 has a chemical formula of C_38_H_23_Cl_2_N_14_Na_5_O_18_S_5_, a molecular weight of 1309.86 and a CAS number of 71872‐76‐9. The open structure of RB 160 is given in **Figure** **13**. Deionized water was used while preparing the aqueous solutions of RB160. The RB160 dyestuff was scanned with a Mapada 1100 series UV spectrophotometer in the range of 400–800 nm, and the wavelength was measured at 620 nm.

### Adsorption Studies

The experiments were carried out in batch mode on an Edmund Bühler Gmbh KS‐15 model shaker at a shaking speed of 225 rpm at determined temperatures by adding the adsorbent to dyestuff solutions at different concentrations in 250 ml capped flasks. At the end of the experiment, each suspension was centrifuged at 5000 rpm for 15 min in a Nuve NF 1215 model centrifuge, and the concentrations of the RB160 solutions at equilibrium were determined by examining the absorbance values of the samples taken from the solutions in certain volumes on a UV spectrophotometer and using a calibration curve. The pH of the solutions was adjusted by adding 0.1 m HCL and 0.1 m NaOH solution, and the solutions were monitored with the Thermo Orion 3 Star model, a digital pH meter. All chemicals used in the experiments were of analytical purity. The values ​​of the parameters such as contact time (min), initial dye concentration (mg L^−1^), temperature (°C), solution pH and adsorbent dosage (g L^−1^) investigated in NGCM adsorption processes are 10‐30‐45‐60*‐65‐70‐75‐80; 50‐100‐150*‐200‐250; 20*‐30‐35‐40; 2‐4‐5.7*‐6‐8‐10 and 10–20*‐30‐40‐50, respectively (*Fixed values ​​selected in the experiments for the effect of parameters).

### Characterizations

Characterization of the NGCM surface was performed using ZEISS SIGMA 300 type field emission scanning electron microscopy (SEM). The NGCMs were characterized via XRD using Rigaku 2200D/max an X‐ray diffractometer equipment with a Cu‐Kα radiation source. A Fourier transform infrared spectroscopy image of RB160 was obtained by scanning in the frequency range 4000–400 cm^−1^ with Bruker Vertex 70v model spectroscopy to determine structural changes and functional groups containing oxygen entering the basal plane (sulfonate, hydroxyl, etc.).

Thermogravimetry/differential thermal analysis (TG‐DTA) was performed on a NETZSCH STA 409 PC Luxx instrument at high resolution. Under atmospheric pressure, the NGCM was analyzed from 20 to 1000 °C at 10 °C m^−1^ in heating rates. The surface areas and pore sizes of the adsorbents were measured using the Brunauer‐Emmett‐Teller (BET) method in the Micrometrics 3Flex instrument, which includes the adsorption‐desorption isotherm of N_2_ at 75.9 K.

### Adsorption Isotherm

To develop mathematical models, the equilibrium data obtained from adsorption experiments and from the removed dyestuff need to be analyzed. Adsorption isotherms are used to accurately describe adsorption for design purposes.

The adsorption isotherm models widely used in aqueous solutions to analyze experimental data include the Langmuir, Freundlich, Temkin, and Dubinin‐Radushkevich (D–R) models.^[^ ^57^ ^]^

### The Langmuir Isotherm

The Langmuir isotherm is applied as single‐layer adsorption from the solution to the surface containing the same region. The adsorption capacity is the maximum amount that covers the adsorbent surface in a single layer. The Langmuir isotherm^[^ ^58^ ^]^ is expressed as Equation (1):

(1) $$\frac{C_{e}}{q_{e}}=\frac{C_{e}}{q_{m}}+\frac{1}{K_{L}\times q_{m}}$$

The Langmuir isotherm is given according to the dimensionless equilibrium parameter (*R*_L_), expressed in Equation (2): The basic properties of a Langmuir isotherm are given according to a dimensionless balance parameter (*R*_L_),^[^ ^59^ ^]^ which is expressed by Equation (2).

(2) $$R_{L}=\frac{1}{1+K_{L}C_{0}}$$

The *R*_L_ values represent the type of isotherm: unfavorable (*R*_L_ > 1), linear (*R*_L_ = 1), favorable (0 < *R*_L_ < 1), or irreversible (*R*_L_ = 0)).^[^ ^60^ ^]^

### The Freundlich Isotherm

The Freundlich isotherm is widely used to describe multilayer sorption as well as nonideal adsorption on heterogeneous surfaces. Adsorption is predicted to occur in regions of different energies. The value of the Freundlich constant (n) varies between 1 and 10 and is given by Equation (3).^[^ ^61^ ^]^

(3) $$\log\left(q_{e}\right)=\log\left(K_{f}\right)+\frac{1}{n}\log\left(C_{e}\right)$$

### The Temkin Isotherm

The Temkin isotherm is used to best explain adsorbate–adsorbent interactions and binding energies. It is expressed in Equation (4).^[^ ^62^ ^]^

(4) $$q_{e}=B_{T}\times \ln\left(K_{T}\right)+B_{T}\times \ln\left(C_{e}\right)$$

where *B*_T_ =  *RT*/*b*_T_.

### The Dubinin–Radushkevich Isotherm

The Dubinin–Radushkevich isotherm is used to estimate the porosity of the adsorbent, the Gaussian energy distribution of a heterogeneous surface and apparent adsorption energies (especially in porous adsorbents). This model equation is given in Equation (5).^[^ ^63^ ^]^

(5) $$\ln\;\left(q_{e}\right)=\ln\left(q_{m}\right)-\beta \times \;\varepsilon^{2}$$

where ε, is the Polanyi potential, $\varepsilon =R_{g}T\ln\left(1+\frac{1}{C_{e}}\right)$.

### Adsorption Kinetics

To determine how dye adsorbs onto the adsorbent surface, the compatibility of several previously developed kinetic models with the experimental data was investigated.

The adsorption kinetic models widely used in aqueous solutions include the pseudo‐first‐order, pseudo‐second‐order, Elovich and intraparticle diffusion models.^[^ ^64^ ^]^ To determine the kinetic models are shown in Equations ((6), (7), (8), (9))

### Pseudo‐First‐Order Kinetic Model

The pseudo‐first‐order model assumes that the adsorption process is controlled by physical diffusion and that equilibrium is reached at a certain rate on the adsorbent surface. The pseudo‐first‐order equation is defined by Equation (6):^[^ ^65^ ^]^

(6) $$\;\log\left(q_{e}-q_{t}\right)=\;\log q_{e}-\frac{k_{1}t}{2.303}$$

here, *q*_e_ is the amount of adsorbed substance per gram of adsorbent at equilibrium (mg g^−1^); *q*_t_ is the amount of substance adsorbed per gram of adsorbent at any given time (mg g^−1^);

*k*_1_ rate constant (min^−1^); *t* is the contact time.

The rate constant *k*_1_ is calculated by graphing log (*q*_e_‐*q*_t_) against t, and the theoretical *q*_e_ value is calculated from its slope and the intersection point of the graph.

### Pseudo‐Second‐Order Kinetic Model

The pseudo‐second order model assumes that in the adsorption process, electrostatic interactions or stronger bonds play an important role on the adsorbent surface. This model was developed by Ho et al. The linear equation is obtained and defined by Equation (7).^[^ ^66^ ^]^

(7) $$\;\frac{t}{q_{t}}=\frac{1}{k_{2}q_{e}^{2}}+\frac{1}{q_{e}}t$$

In this equation, *k*_2_ rate constant (g mg^−1^ min^−1^).

### Elovich Kinetic Model

The Elovich model is a kinetic model that considers variations in activation energy on the adsorption surface and accounts for heterogeneous surface properties. This model is used to describe chemical adsorption kinetics. The Elovich model is defined by Equation (8).^[^ ^67^ ^]^ It is expressed as follows.

(8) $$q_{t}=\frac{\ln\left(\alpha \beta \right)}{\beta }+\frac{\ln t}{\beta }$$

here, *α*: Initial adsorption rate constant (mg g^−1^ min^−1^);

*β*: Desorption constant (g mg^−1^);

*q*_t_: the amount of substance adsorbed at time *t* (mg g^−1^)

If ln(*t*) is plotted against *q*_t_, *β* and *α* are calculated from the slope and shift of the graph, respectively.

### Intraparticle Diffusion Model

The intraparticle diffusion model assumes that the adsorption process in porous materials is controlled by an internal diffusion mechanism. This model is the diffusion hypothesis that occurs through the pore structure developed by Weber and Morris. The intraparticle diffusion model is defined by Equation (9).^[^ ^68^ ^]^

Equation:

(9) $$q_{t}=k_{\mathrm{dif}}\;\sqrt{t}+C$$

here, *k*_dif_: Intraparticle diffusion rate constant (mg g^−1^ min^−2^);

C: An expression of the boundary layer thickness during adsorption;

t: time.

The rate constant *k*_dif_ is calculated from the slope of the graph drawn against *q*_t_ versus *t* ^1/2^. *C* is calculated from the shift of the graph.

### Adsorption Thermodynamics

The Gibbs free energy (Δ*G*°) determines whether the adsorption process will occur spontaneously. If Δ*G*° is positive, adsorption occurs via an external effect; if Δ*G*° is negative, the adsorption process occurs spontaneously without any external influence. ∆*G*° ranges from ‐20 to 0 kJ mol^−1^ during physical adsorption, while it varies from ‐80 to ‐400 kJ mol^−1^ during chemical adsorption.^[^ ^69^ ^]^

According to Equation (10), entropy and enthalpy changes can also be found. If the enthalpy change during adsorption is negative, the reaction is exothermic; if it is positive, the reaction is endothermic.

To determine the thermodynamics parameters, the mathematical expressions for enthalpy change (Δ*H*°, kJ mol^−1^), Gibbs free energy change (Δ*G*°, kJ mol^−1^), and entropy change (Δ*S*°, kJ mol^−1^) are shown in Equations ((10), (11), (12), (13)).^[^ ^70^, ^71^, ^72^ ^]^

(10) $$\;\Delta G^{o}=\Delta H^{o}\;-T\Delta S^{o}$$

The enthalpy value determines whether the adsorption is physical or chemical. According to the literature, a ∆*H*° value of <20 kJ mol^−1^ in the reaction indicates physical adsorption, and a ∆*H*° value of 80–200 kJ mol^−1^ indicates chemical adsorption. ∆*H*° is related to the various interactions that occur in the reaction. The distance between the adsorbent and the adsorbed dye is established.^[^ ^73^ ^]^

(11) $$\Delta G^{\circ }=-R_{g}T\times \ln\left(K_{c}\right)$$

*K*_c_ is the linear sorption distribution coefficient or single point as follows (Equation 12):

(12) $$K_{c}=\frac{q_{e}}{C_{e}}$$

∆*H*° and ∆*S*° were calculated from the following formula (van't Hoff equation) (Equation 13):

(13) $$\ln\left(K_{c}\right)=\left(\Delta S^{\circ }/R_{g}\right)\;-\;\left(\Delta H^{\circ }/R_{g}T\right)\;$$

*R*_g_: Gas constant (8.314 J mol^−1^ K^−1^).

By using these equations, the effect of temperature on the adsorption process of RB 160 onto NGCM was examined.

A preprint of this paper was previously published at Research Square in 2024.^[^ ^74^ ^]^

## Conflict of Interest

The authors declare no conflict of interest.

## Data Availability

Research data are not shared.
